# Extracellular Vesicles and Nanoparticles in Regenerative and Personalised Medicine: Diagnostic and Therapeutic Roles—A Narrative Review

**DOI:** 10.3390/pharmaceutics17101331

**Published:** 2025-10-14

**Authors:** Elena Silvia Bernad, Ingrid-Andrada Vasilache, Robert Leonard Bernad, Lavinia Hogea, Dragos Ene, Florentina Duica, Bogdan Tudora, Sandor Ianos Bernad, Marius Lucian Craina, Loredana Mateiovici, Răzvan Ene

**Affiliations:** 1 Center for Laparoscopy, Laparoscopic Surgery and In Vitro Fertilization, Department of Obstetrics and Gynecology, Faculty of Medicine, Victor Babes University of Medicine and Pharmacy, 300041 Timisoara, Romania; bernad.elena@umft.ro (E.S.B.); mariuscraina@umft.ro (M.L.C.); 2 Center for Neuropsychology and Behavioral Medicine, Victor Babes University of Medicine and Pharmacy, 300041 Timisoara, Romania; hogea.lavinia@umft.ro; 3 Department of Mother and Child Care, “Grigore T. Popa” University of Medicine and Pharmacy, 700115 Iasi, Romania; ingrid-andrada.vasilache@umfiasi.ro; 4 Faculty of Computer Science, Politehnica University of Timisoara, 300006 Timisoara, Romania; robibernad11@gmail.com; 5 Department of Neuroscience, Victor Babes University of Medicine and Pharmacy, 300041 Timisoara, Romania; 6 Bucharest Emergency Clinical Hospital, 014461 Bucharest, Romania; dragos_ene@umfcd.ro (D.E.); florentina.duica@umfcd.ro (F.D.); 7 Department of Surgery, “Carol Davila” University of Medicine and Pharmacy, 050474 Buchurest, Romania; 8 Sigma Evolution, 030167 Bucharest, Romania; bogdani.tudora@gmail.com; 9 Centre for Fundamental and Advanced Technical Research, Romanian Academy—Timisoara Branch, Mihai Viteazul Str. 24, 300223 Timisoara, Romania; 10 Research Center for Engineering of Systems with Complex Fluids, Politehnica University Timisoara, Mihai Viteazul Str. 1, 300222 Timisoara, Romania; 11 Netphen Day Clinic of Wittgenstein Clinic, 57250 Netphen, Germany; loredana.moise93@yahoo.com; 12 Orthopedics and Traumatology Departament, “Carol Davila” University of Medicine and Pharmacy, 050474 Bucharest, Romania; razvan.ene@umfcd.ro

**Keywords:** extracellular vesicles, nanoparticles, regenerative medicine, personalised medicine, hybrid nanoplatforms, artificial intelligence, cytotoxicity, translational barriers

## Abstract

**Background:** Degenerative, metabolic and oncologic diseases are scarcely amenable to the complete reconstruction of tissue structure and functionalities using common therapeutic modalities. On the nanoscale, extracellular vesicles (EVs) and nanoparticles (NPs) have emerged as attractive candidates in regenerative and personalised medicine. However, EV transfection is hindered by its heterogeneity and low yield, while NPs suffer from cytotoxicity, immunogenicity, and long-term safety issues. **Scope of Review:** This review synthesises data from over 180 studies as part of a narrative synthesis, critically evaluating the disease-specific utility, mechanistic insights, and translational obstacles. The focus is laid on comparative cytotoxicity profiles, the capacities of hybrid EV–NP systems to circumvent mutual shortcomings, and the increasing impact of artificial intelligence (AI) on predictive modelling, as well as toxicity appraisal and manufacturing. **Key Insights:** EVs have inherent biocompatibility, immune evasive and organotropic signalling functions; NPs present structural flexibility, adjustable physicochemical properties, and industrial scalability. Common molecular pathways for NP toxicity, such as ROS production, MAPK and JAK/STAT activation, autophagy, and apoptosis, are significant biomarkers for regulatory platforms. Nanotechnological and biomimetic nanocarriers incorporate biological tropism with engineering control to enhance therapeutic efficacy, as well as their translational potential. AI approaches can support rational drug design, promote reproducibility across laboratories, and meet safe-by-design requirements. **Conclusions:** The intersection of EVs, NPs and AI signifies a turning point in regenerative nanomedicine. To advance this field, there is a need for convergence on experimental protocols, the adoption of mechanistic biomarkers, and regulatory alignment to ensure reproducibility and clinical competence. If realised, these endeavours will not only transition nanoscale medicament design from experimental constructs into reliable and patient-specific tools for clinical trials, but we also have the strong expectation that they could revolutionise future treatments of challenging human disorders.

## 1. Introduction

Degenerative, metabolic and oncological disorders are a significant challenge for health systems worldwide. Although several pharmacological, surgical and prosthetic approaches have been developed, they are not yet able to perfectly restore tissue morphology and function [1,2]. This constraint has driven the rise of regenerative and personalised medicine as interdisciplinary fields that integrate stem cell biology, tissue engineering, nanotechnology, and computational sciences to correct or regenerate diseased tissues and restore homeostasis.

### 1.1. Extracellular Vesicles in Regenerative Medicine

Extracellular vesicles (EVs), composed of exosomes (30 to 150 nm), microvesicles (100–1000 nm), and apoptotic bodies, constitute secretory products from most cell types, containing proteins, lipids, messenger RNAs (mRNAs), microRNAs (miRNAs), and long non-coding RNAs (lncRNAs). They are important mediators of angiogenesis (neovascularisation), immune modulation, neuroprotection and tissue repair/regeneration [3].

There is increasing evidence in support of clinical translation:

− Musculoskeletal repair: EVs derived from mesenchymal stem cells (MSCs) augment osteogenesis and chondrogenesis [4].
− Neurovascular regeneration: EVs from dental pulp promote angiogenesis and neurogenesis in ischemic models [5].
− Wound repair: Vascular endothelial growth factor (VEGF) and transforming growth factor-beta 1 (TGF-β1)-overexpressing EVs lead to enhanced wound closure and vascularisation, depending on the microenvironment [6].
− Neurodegenerative biomarkers: phosphorylated tau and α-synuclein—loaded circulating extracellular vesicles for the differential diagnosis of Alzheimer’s and Parkinson’s disease [7].

EVs can cross the blood–brain barrier (BBB) and exhibit low immunogenicity, both of which are favourable characteristics for systemic administration. However, their heterogeneity, low yield and nonstandardized isolation and manufacturing processes remain challenging [8].

### 1.2. Nanoparticles in Regenerative Medicine

Nanoparticles (NPs), generally ranging from 1 to 200 nm, represent an engineered counterpart of EVs, as their physicochemical properties (size, surface charge, morphology, and functionalization) can be accurately tuned to influence biodistribution and cellular uptake, as these parameters regulate the interaction between NPs and cells [9,10].

- Metallic NPs: Silver nanoparticles (AgNPs) are antimicrobial, gold nanoparticles (AuNPs) and copper oxide NPs are used for photo-thermal and imaging technology [11,12].
- Magnetic NPs: Iron oxide nanoparticles (IONPs) enable delivery and theranostic imaging facilitated by an external magnetic field in cardiovascular regeneration [13,14,15].
- Polymeric NPs: U.S. Food and Drug Administration (FDA)–approved, poly (lactic-co-glycolic) acid (PLGA) and polyethylene glycol (PEG), a biodegradable NP with an excellent safety profile [16,17].
- Lipid-based NPs: Liposomes and micelles are well-established carriers for therapy [18].

However, even though metallic and oxide NPs can elicit reactive oxygen species (ROS) generation, mitochondrial stress, apoptosis, and activation of inflammatory cascades through mitogen-activated protein kinase (MAPK) and Janus kinase/signal transducer/activator of transcription (JAK/STAT) pathways, even biodegradable polymers trigger complement activation or immune responses on some occasions [19,20].

### 1.3. Translational Barriers and Emerging Solutions

The reciprocal shortcomings of EVs and NPs highlight three overriding translational challenges:

1. Toxicity—as associated with ROS production, apoptosis, and immune stimulation.
2. Immunogenicity—mediated by protein corona development and complement activation.
3. Scalability—limited by EV diversity and NP production repeatability.

To address these issues, several hybrid and biomimetic systems have been investigated:

(a) Membrane-coated NPs exploit native surface proteins for immune evasion and tissue targeting [21].
(b) Engineered biomimetic particles (EBPs) replicate EV morphology but ensure scalability [8].
(c) EV–NP fusion hybrids combine biological tropism with synthetic multifunctionality [6,22].

These platforms aim to bridge the gap between the biological specificity of EVs and the engineered control of NPs, thereby resolving translational impediments.

### 1.4. Role of Artificial Intelligence

The potential of predictive integrated approaches revolves around artificial intelligence (AI). Machine learning (ML) and deep learning (DL) have been used to:

- Predict from size, zeta potential, and chemical composition descriptors of nanotoxicity [23,24].
- Classify EV cargo and morphology for cancer diagnosis, using Convolutional Neural Networks (CNNs) [25,26].
- Maximise production and reproducibility in EV loading and NP design using AI-based process modelling [27,28].

In that regard, AI is not only a discovery tool but also a regulatory and translational connector, enabling clinical adoption.

### 1.5. Aim of the Review

In this review, a critical and integrative analysis is made of nanoscale therapeutics in regenerative and personalised medicine. It specifically:

i. Considers EV- and NP-based therapies in the context of disease.
ii. Explores mixed hybrid and biomimetic systems combining natural inspiration with synthetic advancements.
iii. Investigates the implications of AI in predictive design and personalised application.
iv. Conducts a critical review of cytotoxicity and safety, providing systematic comparisons across classes of NPs.

Based on more than 145 studies, this review aims to establish a rigorous roadmap for clinical translation, connecting innovation with safety, scalability, and personalisation.

## 2. Methodology

We prepared and reviewed this paper as a narrative review to summarise the findings on EVs and NPs in the field of regenerative and personalised medicine. A narrative approach, in contrast to the strict selection criteria generally used for systematic reviews, allows for the integration of various forms of evidence, including experimental reports, translational studies, and review articles—along with the critical interpretation and contextualization required for clinical translation.

### 2.1. Literature Search Strategy

A comprehensive literature search was conducted in PubMed, Scopus, and the Web of Science Core Collection, covering the period from January 2010 to August 2025. Additional references were identified through backward and forward citation tracking of key articles. Search terms and Boolean combinations included:

− “extracellular vesicles” OR “exosomes” OR “microvesicles” AND “regenerative medicine”
− “nanoparticles” OR “nanomedicine” AND “personalised medicine”
− “biomimetic platforms” OR “hybrid nanoparticles” AND “therapy”
− “artificial intelligence” OR “machine learning” AND “nanoparticle design” OR “EV diagnostics”
− “cytotoxicity” OR “safety profiles” AND “nanoparticles” OR “extracellular vesicles”

**Figure 1:**
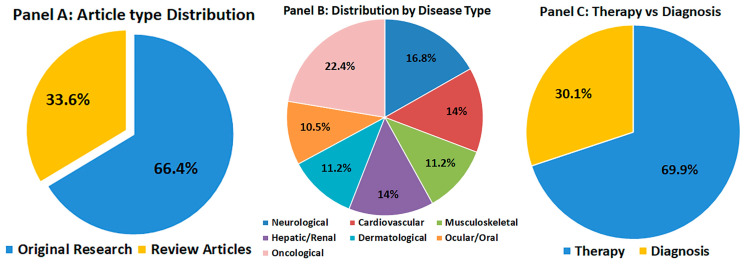
Overview of the 145 studies included in this review. (**A**) Distribution of article types (original research vs. review articles). (**B**) Distribution by disease type: neurological, cardiovascular, musculoskeletal, hepatic/renal, dermatological, ocular/oral, and oncological disorders. (**C**) Proportion of studies focusing on therapeutic versus diagnostic applications. This visualization highlights the scope, thematic diversity, and translational balance of the included literature.

As illustrated in Figure 1, the screened literature demonstrates a balanced coverage across disease categories and a predominance of therapeutic over diagnostic applications, reinforcing the translational focus of this review.

#### 2.1.1. Inclusion and Exclusion Criteria

Inclusion criteria: peer-reviewed articles (original research, preclinical, clinical reports and reviews) concerning diagnostic or therapeutic use of EVs and NPs for regenerative medicine, oncology, cardiovascular diseases (CVD), neurology, infectious disease or musculoskeletal pathology. Articles in English published in the past 15 years were given preference.

Exclusion criteria: conference abstracts with no full-text available, non-peer-reviewed sources, and studies on purely non-biomedical nanomaterials (e.g., environmental or industrial nanostructures).

In this paper, we intentionally limited our examination to mammalian extracellular vesicles (EVs), as these types of vesicles are currently the most well-studied and clinically relevant platforms in regenerative and personalised medicine. Mammalian EVs, such as those from mesenchymal stem cells, immune cells, or progenitor cells, also have many mechanistic studies demonstrating that they can cross biological barriers, modulate immunity, and transfer bioactive RNA-protein cargo [29,30,31]. Furthermore, such vesicles have already been advanced to preclinical and early clinical studies in cardiovascular, neurological, and musculoskeletal diseases, demonstrating the immediate translational relevance [32,33].

In comparison, EVs from plants and microbes, despite growing interest in drug delivery and immunomodulation purposes, are still in the early stages of investigation. Although plant and microbial EVs are being considered as stable oral delivery vehicles and possible mediators of host-microbiome interaction, they still lack a thorough understanding of their mode of action, options for scalability, or a safety profile to be integrated within translational frameworks comparable to those existing for mammalian EVs. Therefore, their specific discussion was considered beyond the objectives of the present review.

Therefore, due to the efficiency of targeting mammalian EVs specifically, this narrative offers evidence that can be immediately utilised in preclinical modelling, regulatory considerations, and patient-specific interventions for the translation of the work into a clinical setting [29,30,31]. Simultaneously, it is also pertinent to note that the use of plant- and microorganism-derived EVs has recently emerged as a promising area in EV research. However, future dedicated reviews are needed when their biological features and therapeutic potentials are more clearly defined.

#### 2.1.2. Data Extraction and Synthesis

Based on the search, about 145 pertinent articles were found. Extracted data focused on:

(a) EV isolation methodologies, biological origin and function.

(b) HM physicochemical properties (size, charge, coatings, functionalization).

(c) Specific therapeutic and diagnostic outcomes of patented diseases.

(d) Safety and cytotoxicity results.

(e) New strategies for hybrid biomimetic systems and AI-guided nanomedicine.

Thematic interpretation was condensed into four major categories:

1. Disease-specific regenerative applications.
2. Hybrid and biomimetic platforms.
3. Artificial intelligence in nanomedicine.
4. Cytotoxicity and safety profiles.

We adopted a narrative review method to focus on comparisons across methods, critical interpretation of results, and translational implications, which are often neglected in strict systematic analyses.

#### 2.1.3. Limitations of the Methodology

As a narrative review, this approach has its limitations. The lack of a systematic method may lead to selection bias, although the selected studies were based on topic and scientific contribution, and do not represent an exhaustive summary of all available literature. Limitations to English language publications may have resulted in the loss of relevant data in other languages. Furthermore, the use of in vitro or in vivo studies and early clinical reports varies among articles, making direct comparison difficult. Unlike a systematic review or meta-analysis, the study does not perform a quantitative synthesis of effect sizes.

Nonetheless, the narrative format allowed us to incorporate diverse evidence, emphasise mechanistic insights and point to translational challenges, thus providing an integrated and critical overview that fits well with the emerging interdisciplinary nature of regenerative nanomedicine.

## 3. Regenerative Medicine by Disease Type

### 3.1. Immunogenicity Differences Between EVs and NPs

#### 3.1.1. Immunogenicity of EVs

EVs generally exhibit low immunogenicity due to their natural composition, which mimics the cell membrane. This property allows EVs to evade immune recognition and persist longer in circulation [30,31]. However, the immunogenicity of EVs can be influenced by their cellular origin and the presence of specific surface antigens. For instance, EVs derived from immune cells, such as dendritic cells, may exhibit higher immunostimulatory potential compared to EVs from mesenchymal stem cells [29].

#### 3.1.2. Immunogenicity of NPs

In contrast, synthetic NPs often exhibit higher immunogenicity due to their non-biological composition and surface properties. The immune system can recognise NPs as foreign particles, leading to their rapid clearance by phagocytic cells [32,34]. This immune response can limit the therapeutic efficacy of NP-based delivery systems. However, strategies such as surface modification with biocompatible materials or targeting ligands can reduce the immunogenicity of NPs and improve their delivery efficiency [33,34]. A comparative analysis of EVs and NPs is summarised in Table 1.

### 3.2. Neurological and Neurodegenerative Disorders

The central nervous system (CNS) has a limited inherent capacity for repair and has therefore been at the forefront of restorative treatments. EVs of mesenchymal stem cells (MSC-EVs) contain microRNAs (miRNAs), including miR-124 and miR-21, which are implicated in the downregulation of apoptotic genes, increase neuronal survival, and promote synaptic reorganisation [6,40,41,42,43,44,45,46]. In ischemic rodent models of stroke, EV therapy has been shown to reduce infarct volume and motor functional recovery [38,41]. EVs also enhance angiogenesis in ischemic brain tissue by transferring vascular endothelial growth factor (VEGF) and hypoxia-inducible factor 1-alpha (HIF-1α) [43,44].

Engineered NPs complement these effects. Poly(lactic-co-glycolic acid) (PLGA) and lipid NPs are ligand decorated, i.e., transferring to transverse the BBB [47]. They are released upon delivery to trigger antioxidant or neurotrophic activity, decrease the levels of reactive oxygen species (ROS), and restore mitochondrial function [48,49]. These tactics illustrate that EVs hold biologically active payloads and NPs can achieve site-specific targeting beyond the neuroprotection barriers.

### 3.3. Cardiovascular Disease

The heart after myocardial infarction is susceptible to fibrosis and contractile tissue loss. Cardiac progenitor cell-derived EVs (CPC-EVs) and MSC-EVs transfer angiogenic factors, such as VEGF, HIF-1α, and cardioprotective ncRNAs, to induce endothelial cell proliferation and migration [41,50,51,52]. They decrease the apoptosis of cardiomyocytes and enhance ventricular remodelling in preclinical models [52].

On the synthetic side, PEG and PLGA NPs encapsulating growth factors or drugs (e.g., statins) are released locally in the ischemic area [53,54]. This enhances myocardial perfusion without causing side effects in the systemic circulation. Hybrid EV–NP platforms are being analysed to harness the advantages of paracrine signalling by EVs and the sustained release kinetics offered by NPs [55,56,57].

### 3.4. Musculoskeletal Disorders

Bone and cartilage healing requires not only osteogenic promotion but also the suppression of inflammation. Osteoblast and chondrocyte EVs activate the Wnt/β-catenin pathway, promote mineralisation, and decrease pro-inflammatory cytokines (tumour necrosis factor-alpha (TNF-α) and interleukin-1 beta (IL-1β)) [58,59,60,61,62]. MSC-EVs promoted cartilage thickness and mechanical stability in osteoarthritis models [8].

Hydroxyapatite (HA) NPs, silica NPs and polymeric carriers offer osteoconductive scaffolding, while conjugation of bone morphogenetic protein-2 (BMP-2) promotes bone healing [22,53,54,63,64,65,66,67,68,69,70,71]. NP-modified scaffolds mimic the extracellular matrix (ECM) environment and form a 3D structure suitable for the growth of osteoblasts and chondrocytes. Therefore, EVs are biologically active signals, and NPs offer structural and pharmacological support.

### 3.5. Hepatic and Renal Disorders

Hepatocyte- and MSC-derived EVs promote liver regeneration by suppressing the transforming growth factor-β (TGF-β)/Smad signalling pathway, attenuating fibrogenesis, and enhancing hepatocyte proliferation [6,8,21,72]. Enriched in antioxidant enzymes, for example, superoxide dismutase (SOD) and catalase, EVs alleviated tubular cell apoptosis with improved renal function in a murine model of renal ischemia–reperfusion injury [8], whereas they have also been shown to attenuate M2 macrophage-mediated inflammation [50].

The polymeric NPs enable the delivery of antifibrotic compounds, siRNAs, or hepatoprotective molecules specifically to the damaged tissue [40,73]. Metallic NPs, especially iron oxide-based ones, are effective as imaging agents but not acceptable for therapy due to their potential to induce oxidative stress. Hence, EVs exert paracrine regenerative effects, and polymeric NPs allow for safe pharmacological treatment.

### 3.6. Skin and Wound Healing

The skin is also one of the organs most readily treated by EVs. Fibroblast- and keratinocyte-derived EVs promote wound healing by stimulating angiogenesis, collagen accumulation, and keratinocyte migration [8,38,41,42,43,44,45,46]. EVs loaded with miR-21 result in the downregulation of pro-inflammatory cytokines and the promotion of vascularisation, which is observed in diabetic wounds where such EVs have been applied [38,42,43].

NPs, especially silver nanoparticles (AgNPs) and zinc oxide nanoparticles (ZnO NPs), are used in wound dressings as antibacterial agents [37,74,75,76,77]. However, the long-term harm limits their use to low dosages. Hybrid EV–NP wound dressings are one such solution, in which the material based on EVs promotes tissue repair, while the NPs help control localised microbial contamination.

### 3.7. Ocular, Oral, and Cardiac Tissue Engineering

In dentistry, EVs derived from dental pulp stem cells (DPSCs) induce odontoblast-like cell differentiation and angiogenesis, supporting pulp regeneration [22,53,54,63,64,65,66,67,68,69,70,71]. In ophthalmology, EVs promote the migration of corneal epithelial cells, inhibit inflammation, and contribute to wound healing of the cornea after injury.

The NP-based scaffolds mimic the ECM, promote the biomechanical properties of engineered tissues and provide a controlled release of growth factors. In myocardial tissue engineering, EV-embedded scaffolds enhance vascular integration and grafted cell survival, whereas NPs reinforce the scaffold’s structure and provide imaging ability.

### 3.8. Oncology and Post-Therapy Regeneration

Although cancer treatment is not regenerative, the healing of tissue after surgery and radiotherapy has a significant overlap. Stromal cell-derived EVs normalise vascular and immune function in irradiated organs [8,38,41,42,43,44,45,46]. NPs help by carrying anticancer drugs in a local manner, which decreases systemic toxicity and spares tissue regenerative potential [73,78]. Theranostic NPs that already demonstrate imaging and therapeutic properties can be used for monitoring tissue repair during cancer therapy.

### 3.9. Summary

Similar effects can be observed when stratified by disease type:

1. Neurology: EVs offer neuroprotection and angiogenesis, while NPs facilitate BBB crossing and controlled release.
2. Cardiology: EVs decrease apoptosis and promote angiogenesis; polymeric NPs support the delivery of cardioprotective agents.
3. Musculoskeletal: EVs promote osteogenesis and chondrogenesis; HA- and polymeric NPs help with scaffolds and growth factor delivery.
4. Hepatic/Renal: EVs reduce fibrosis and apoptosis; polymeric NPs for safe targeted delivery.
5. Skin: EVs accelerate the speed-up power closure and angiogenic process, while AgNPs and ZnO NPs provide an additional antimicrobial armour.
6. Other tissues: EVs versus NPs ECM-mimetic scaffolds for dental, ocular and myocardial regeneration. EVs and NPs may have better efficacy when used in combination.
7. Oncology overlap: EVs repair tissue homeostasis; NPs enable targeted therapy and theranostics.

On all platforms used, EVs prove to be safe and regeneratively potent components, whereas NPs contribute precision, tuneability and structurality. Polymeric carriers are still the most clinically applicable among NPs. Metal nanoparticles are well-suited for antimicrobial or imaging applications, where controlled dosage can reduce their cytotoxicity. In the future, standardisation of readouts will be beneficial, as will integration with mechanisms (ROS, TGF-β, Nrf2, MAPK, and JAK/STAT), and creating hybrid EV–NP systems optimised to treat each disease.

## 4. Hybrid and Biomimetic Platforms

Both EVs and NPs have made significant contributions to the development of nanomedicine; however, their clinical potential is limited due to counterbalancing limitations. EVs are superior in biocompatibility, intrinsic targeting, and molecular communication; however, they remain challenging due to their heterogeneity, as well as the low yield and complexity of producing them at a large scale [8,21]. On the other hand, NPs offer tunable design, reproducibility, and industrial scalability, but are limited by immune clearance, protein corona formation, and possibly cytotoxicity [17,40,79,80,81,82,83,84,85]. Hybrid and biomimetic systems are an expanding strategy that aims to merge the biological properties of EVs with the engineering flexibility of NPs, enabling multifunctional nanocarriers with immune evasion, targeted delivery, and controlled release for regenerative and personalised medicine.

### 4.1. Membrane-Coated Nanoparticles

Nanoparticles coated with the cell’s own membrane utilise surface proteins, glycans, and antigenic signatures from its original cell of origin, allowing the synthetic core to escape immune clearance and re-homing in specific tissues [21].

− Immune evasion: Red blood cell (RBC)-derived coatings express CD47 and other “self-markers,” which inhibit phagocytosis by macrophages, thereby prolonging systemic circulation [8,21].
− Targeting damaged vasculature: Encapsulation of platelets (PLT) into a membrane increases their adhesion potential to sites of vascular injury/inflammation, favouring delivery into ischemic tissue or stented vessels [86].
− Antimicrobial use: By inducing platelets to coat NP, this also allows for the targeting of bacterial colonised biofilms and, therefore, localised antimicrobial therapy at infection sites [79,80,87].
− Oncology: Tumour-membrane-coated NPs retain adhesion molecules, including integrins and cadherins, facilitating homotypic recognition and selective enrichment within primary/metastatic tumours [8,21]. This has led to better delivery of both chemotherapeutic and imaging drugs [4,5,88].
− Immune cell mimicry: Macrophage and leukocyte membrane coatings modulate NP migration toward inflamed tissue, favouring targeted delivery of anti-inflammatory or immunomodulatory agents [81,82,83].

These biomimetic systems combine target selectivity with a long half-life and immune avoidance, which brings significant value to oncology, infectious diseases, and cardiovascular regeneration.

### 4.2. Engineered Biomimetic Particles (EBPs)

The engineered biomimetic particle (EBP) mimics the shape of the EV but is synthesised for reproducibility and scalability [8].

− Tailored physicochemical properties: EBPs can have well-defined size ranges (50–200 nm), optimal zeta potentials, and tunable lipid or polymer shells, enabling batch-to-batch reproducibility [8].
− Cargo loading: EBPs can be designed to load various types of drugs, including siRNA, mRNA, proteins, growth factors, and small-molecule drugs. Furthermore, a host of therapeutic agents have been reportedly encapsulated with drug loading efficiencies that frequently exceed those of natural, bio-derived EVs [8].
− Targeted functionalization: surface modification with antibodies, peptides, or aptamers provides disease-specific tropism, enabling devices to exhibit flexibility beyond the natural homing capabilities of EVs [89,90].
− Stimuli sensitivity: Genchi et al. [22] highlighted the use of EBPs embedded in photo-, magnetically, and acoustically sensitive platforms, enabling spatial and temporal release, to enhance angiogenesis, osteogenesis, and neurogenesis in tissue engineering.
− Translational potential: EBPs mitigate yield and heterogeneity associated with natural EVs while preserving beneficial biological activity, thereby closing the gap between reproducibility and functionality.

### 4.3. EV–NP Fusion Hybrids

Combining EV membranes with NP cores yields chimeric platforms that combine the tropism of biological EVs with the multifunctionality of synthetic NPs [21].

− Drug stability and preservation: EV–NP hybrids enhance encapsulation and protect drugs from enzymatic degradation, thereby prolonging the therapeutic effect [8].
− Theranostic ability: By co-encapsulating imaging probes (fluorescent dyes, magnetic nanoparticles, or radionuclide tracers) and therapeutic drugs, the hybrids enable simultaneous diagnosis and treatment (theranostics) [7].
− Improved biodistribution: These systems maintain the cell-specific targeting ligands of the EV while leveraging NP tuning (size, charge, and ligand conjugation). Therefore, biodistribution can be more consistent and effective compared to synthetic NPs only [89,90].
− Pre-clinical evidence: The preclinical work using MSC-EVs fused with polymeric or metallic cores results in favourable homing to ischemic tissues, augmented angiogenesis, and extended retention in regenerative models [22,89].

### 4.4. Translational and Technological Advantages

Multifunctional nanoplatforms combining hybrid and biomimetic features for overcoming these translational barriers of nanomedicine:

(a) Immune evasion and circulation time: The mobilisation of endogenous membrane cloaks reduces reticuloendothelial system clearance [8,21].
(b) Reproducibility and upscaling: EBPs, as well as NP fusion constructs, can be produced in a reproducible manner under standardised conditions, unlike natural EVs [8].
(c) Multi-functionality: Hybrids combine targeting, therapy, and imaging capacity into a single carrier [7,8,21].
(d) Stimuli responsiveness: External stimuli (light, magnetism, ultrasound) permit controlled release in regenerative scaffolds [22,89].
(e) Decreasing cytotoxicity: The encapsulation or coating of NPs decreases direct contact between reactive surfaces and cells, thereby reducing levels of oxidative stress and inflammation [40,79,80,81,82,83,84,87].

Clinical relevance:

(a) Tumour Membrane-coated NPs and EV–NP hybrids have been utilised for their ability to enhance tumour penetration, theranostic imaging, and localised drug release [4,7,72,86,88].
(b) Infectious disease: Platelet- and leukocyte-coated NPs demonstrate antimicrobial and anti-inflammatory effects [79,80,81,82,83,84,85,87].
(c) Regenerative medicine: EBP and EV–NP hybrids delivered using scaffolds induce the progression of angiogenesis, osteogenesis, and neurogenesis in tissue engineering approaches [22,89,90].

### 4.5. Outlook

As reported by Lorite et al. [21], Piffoux et al. [8], and Genchi et al. [22], hybrid and biomimetic approaches are becoming the frameworks of translational nanomedicine. Combining the evasiveness of the immune system and the tropism of EVs, with the tunability and reproducibility of NPs, these platforms provide a compromise between safety precautions and maintaining scalability and potential efficacy.

Future directions will likely include:

i. Integration with AI: Predictive modelling of biodistribution, immune recognition, and therapeutic response across the hybrid system (as in Chapter 5).
ii. Standardisation: Development of Good Manufacturing Practice (GMP) compliant protocols for consistent manufacturing.
iii. Personalisation: Tailoring hybrids using patient-derived EVs or disease-specific ligands for increased safety and efficacy.

**Figure 2:**
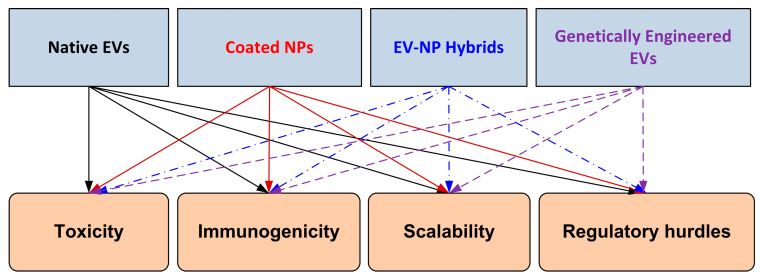
Schematic representation of hybrid extracellular vesicle (EV)/nanoparticle (NP) design strategies and associated translational barriers. The four leading platforms—native EVs, coated nanoparticles, EV–NP hybrids, and genetically engineered EVs—are illustrated at the top. These are mapped to key translational barriers (toxicity, immunogenicity, scalability, and regulatory hurdles) shown at the bottom. The figure highlights the balance between innovation in hybrid design and the challenges that must be overcome for successful clinical translation.

As illustrated in Figure 2, four major hybrid design strategies can be distinguished: native EVs, coated nanoparticles, EV–NP hybrids, and genetically engineered EVs. Each platform presents specific translational barriers that must be addressed, including toxicity, immunogenicity, scalability, and regulatory hurdles. This schematic provides a visual overview of the balance between innovation in hybrid design and the challenges that must be overcome for successful clinical translation.

Therefore, hybrid and biomimetic nanoplatforms serve as a translational intermediate platform, bridging the gap between biological naturalness and synthetic versatility, which can facilitate processes in oncology, infectious disease, and regenerative medicine.

## 5. Artificial Intelligence in Nanomedicine

Artificial intelligence (AI) is emerging as a crucial tool in medical nanotechnology for addressing several long-standing challenges associated with the clinical translation of extracellular vesicles (EVs) and nanoparticles (NPs). Both technologies hold great potential in regenerative and personalised medicine; however, they still suffer from process heterogeneity, scale-up performance variability, and safety issues. AI leverages information-rich datasets derived from multi-omics, high-resolution imaging, and clinico-pathological studies to predict the performance of nanomaterials, optimise their design, and enhance their reproducibility.

### 5.1. AI’s Role in EVs’ Engineering

EVs are small vesicles released by almost all types of cells, and carry proteins, lipids and nucleic acids. They are well-characterised for their diagnostic and therapeutic potential; however, batch-to-batch heterogeneity, production yields, and isolation methods hinder their clinical utility. Three categories are used to test the proposed AI models:

− Diagnostics and classification: Machine learning (ML) combines proteomic and transcriptomic EV profiles to classify diseases. Greenberg et al. [25] demonstrated that ML classifiers can enhance diagnostic accuracy across multiple cancers. Serretiello et al. [26] utilised convolutional neural networks (CNNs) to classify breast cancer subtypes based on EV morphology and microstructure. These methods emphasise the potential of AI in developing non-invasive, reproducible diagnostic devices.
− EV biomarker interpretability: Explainable AI (XAI) tools address transparency by attributing feature contributions within complex EV datasets. Trifylli et al. [91] applied XAI to staging liver disease, identifying which molecular signatures are necessary for predictability.
− EV optimisation for therapy: In the work by Li et al. [27] and Chen et al. [28], predictive models were used to suggest ideal loading conditions for therapeutic molecules into EVs. This will enhance the reproducibility of EV-based treatments, which have been at the forefront of translating complex diseases into effective therapies.

In addition to classification, AI was used for EV isolation optimisation, including the standardisation of parameters for ultracentrifugation and size exclusion chromatography. This is in accordance with the MISEV (Minimal Information for Studies of Extracellular Vesicles) criteria, which focus on the reproducibility and transparency of the data.

### 5.2. AI in Nanoparticle Design

NPs, such as polymeric carriers (polyethene glycol (PEG), polylactic-co-glycolic acid (PLGA)), metallic NPs, and carbon-based nanomaterials, can be tailored with varying sizes, shapes, and charges, along with surface modifications. However, in this case, the parameter space is prohibitively ample for trial and error. AI enhances the rational design by connecting physicochemical descriptors to biological response:

(1) Predictive modelling of toxicity and biodistribution: Yousaf [92], Ahmadi et al. [23], and Yazdipour et al. [24] show that size, charge, and surface chemistry can accurately predict NP toxicity using support vector machines (SVMs), random forests (RFs) and artificial neural networks (ANN). These models minimise the need for animal testing and enhance pre-clinical safety evaluation.
(2) Treatment optimisation: Kapoor et al. [93] developed deep learning and hybrid models of NP–drug interactions to predict tumour accumulation and BBB penetration. Such an approach expedites the development of NPs in cancer and neurological disorders.
(3) Reproducibility of the formulation: Khokhlov et al. [94] demonstrated that AI-driven formulation models can minimise the issue of inter-laboratory variabilities, which remains a significant bottleneck in NP development. It is because AI can associate input conditions (temperature, solvent, ligand ratios) with NP quality that scale-up can be achieved in a standardised manner.
(4) Nano–bio interactions: AI has also been used to simulate NP interactions with the immune system and protein corona, aiming to forecast opsonisation and clearance kinetics [95,96,97]. This assists in predicting immunogenicity and optimising NP stealth techniques.

Combined, these reports demonstrate AI’s ability to accelerate and strengthen the development of safe-by-design nanoparticles that offer both therapeutic efficacy and predictable biodistribution.

### 5.3. The Role of Integrated AI in Nanomedicine

AI embedding in EV and NP study is leading a transformation to precision nanomedicine:

(1) Hybrid EV–NP systems: AI models predict loading and unloading kinetics for cargo(s); targeting efficiency in engineered hybrids, e.g., EV-coated NPs. Models like these reduce cost and time by iteratively optimising design prior to in vivo testing.
(2) Clinical trial simulation: AI can model clinical trial outcomes using patient datasets and pre-clinical EV/NP performance data, thus optimising patient selection and adaptive dosing scenarios.
(3) Regulatory science: While AI’s predictive tools can comply with regulations, they also enable the early detection of toxicity and reproducibility across laboratories. This is especially necessary for companies that require AI explainability and traceability.
(4) Data harmonisation: Standardised EV isolation and NP characterisation protocols are still lacking, representing a significant bottleneck in the field. Artificial intelligence requires consistent datasets for practical training and emphasises the need for multi-centre validation/harmonisation under MISEV guidelines.

### 5.4. AI Applications in EV and NP Studies

To provide an overview of the emerging compounds in AI for nanomedicine, Table 2 highlights recent AI methods, their applications in EV/NP research, and the finalised outcomes.

### 5.5. Summary

AI omits heterogeneity, design complexity and reproducibility limitations in EVs and NPs studies. When machine learning (RF, SVM, and ANN) is coupled with advanced deep learning models (CNNs, hybrid methods), it enhances diagnostics, optimises therapy rapidly, and facilitates safer translation of nanoplatforms. Its uses vary widely, from predicting toxicity and optimising cargo to simulating hybrid EV–NP and designing clinical trials.

As evidenced by a series of recent reports [23,24,25,26,28,91,92,93,94,95,96,97], AI-driven nanomedicine is advancing rapidly from preclinical investigation to clinical translation. By integrating it into (MISEV guidelines-aligned) standardisation activities and regulatory frameworks, the AI-nano may become a cornerstone in regenerative and personalised medicine.

### 5.6. How AI Addresses Traditional Challenges in EV Research

There are four principal challenges traditionally encountered in extracellular vesicle studies:

(1) Heterogeneity of EV populations: The composition (size, content, and cell of origin) of EVs varies, which makes it difficult to compare results between laboratories.
  - AI solution: Machine learning can partition EVs into functional subtypes using high-dimensional data to obtain consistent grouping, thereby reducing heterogeneity [25,26,91].
(2) Poor yield and lack of uniformity in isolation techniques—Ultracentrifugation, precipitation, and chromatography frequently yield various EV profiles.
  - AI solution: Autonomous (AI-driven) optimisation of isolation parameters resulting in standardised radioactivity output and purity, assuring reproducibility, and meeting MISEV guidelines [28].
(3) Clinical translation: Limited reproducibility—Small datasets and variable patient responses prevent reproducible results.
  - AI solution: Multi-omics EV data and patient clinical profiles are integrated for such purposes, increasing the reproducibility of diagnosis and predicting therapy response as well [91].
(4) Poor interpretability of EV biomarkers: Even when predictive, much is unknown about the source and meaning of biomarkers.
  - AI solution: A XAI highlights which of the EV cargo molecules are driving a diagnostic or prognostic prediction, rendering the results understandable to clinicians and regulators [91].

The absence of methodological standardisation remains a significant obstacle in EV research, as diverse isolation and characterisation approaches frequently result in significant variance in yield and composition. To standardise the field, the International Society for Extracellular Vesicles (ISEV) has published consensus recommendations in its MISEV2018 guidelines [98] and their recent revision, MISEV2023 [98]. These guidelines propose minimum information for reporting experiments based on the isolation of EVs, including their composition, concentration, and bioactivity; procedures for measuring their size and granularity; as well as standards for determining the source cells. Moreover, compatibility with these standards not only enhances reproducibility across studies (and algorithms) but also grounds the training of AI models that can accurately identify and distinguish pathological tissues, as they rely on standardised and harmonised datasets.

Once resolved, AI can turn EVs from promising yet erratic research tools into reliable clinical diagnostic, therapeutic and patient monitoring platforms. This part provides a strong foundation for those unfamiliar with the EV space, explaining how AI brings value.

## 6. Cytotoxicity and Safety Profiles

Nanoparticles (NP) are widely investigated for diagnostic, drug delivery, and regenerative needs, but their clinical application is limited due to cytotoxic effects or hypersensitivity responses inherent to sub-optimal blood pool clearance. While EVs are natural and generally compatible, synthetic NPs may cause side effects due to their composition, size, charge, surface chemistry, and context of exposure [99,100,101,102,103,104].

Toxicity is frequently associated with the production of reactive oxygen species (ROS), mitochondrial impairment, oxidative stress response, apoptosis, autophagy, and inflammatory signalling pathways, such as the mitogen-activated protein kinase (MAPK) and Janus kinase/signal transducer and activator of transcription (JAK/STAT) pathways [105,106,107,108,109,110,111,112]. A thorough understanding of these mechanisms is crucial for design optimisation and ensuring clinical safety.

Inclusion Criteria

The cytotoxicity review conducted here includes exclusively published, peer-reviewed original studies (2010–2024) that present:

(1) NP formulations (Composition, Size, Charge variation and surface modifications).
(2) Other established models in vitro (human/rodent hepatocytes, renal epithelial cells, fibroblasts) or in vivo (murine/rodent animal models).
(3) Mechanistic endpoints determined: ROS generation, mitochondrial membrane potential, caspase activation, inflammatory cytokines (IL-6, TNF-α), autophagy (mTOR signalling), and DNA damage.

### 6.1. Metal Nanoparticles

Silver NPs (AgNPs): Some of the most cytotoxic. Size really does matter with regard to toxicity; ultrasmall AgNPs (1.4 nm) produce more ROS, mitochondrial damage and apoptosis than larger particles (18 nm) [11,113,114]. Mechanisms include:

− Formation of adducts with SH groups of the enzymes of the respiration chain→mitochondria damage.
− DNA damage: ROS-mediated fragmentation of the DNA.
− BBB (blood–brain barrier) crossing and neuronal accumulation, resulting in neurotoxicity [115,116,117,118,119].

Gold nanoparticles (AuNPs): Nontoxic in general but known to accumulate within neural tissues (cortex, hippocampus, choroid plexus) upon multiple dosing [19,120]. Mechanisms:

− Production of ROS by activation of nicotinamide adenine dinucleotide phosphate (NADPH) oxidase [110,111].
− Autophagy through mechanistic target of rapamycin (mTOR) signalling [121,122,123].
− Prolonged exposure → neuroinflammatory response [18].

### 6.2. Metal Oxide Nanoparticles

(a) Zinc oxide nanoparticles (ZnO NPs): ZnO NPs are toxic at concentrations exceeding 10 µg/mL, resulting in damage to hepatocytes and renal epithelial cells [124]. Mechanisms:
  − ROS accumulation.
  − Collapse of mitochondria and apoptosis.
  − Inhibition of nuclear factor erythroid 2-related factor (Nrf2)/heme oxygenase-1 (HO-1) antioxidant defensive mechanism [125,126,127].
  − Increased toxicity in response to ultraviolet B (UVB) via MAPK activation [128,129].
(b) Copper oxide nanoparticles (CuO NPs): They are found to be more toxic than ZnO and cause high levels of ROS production, apoptotic index, and release of inflammatory cytokines in neurons and epithelial models [11].
(c) Iron oxide nanoparticles (Fe_3_O_4_, Fe_2_O_3_): Most used as contrast agents and drug carriers, safe at clinical concentrations. Nevertheless, lysosomal storage can lead to pro-inflammatory cascades. The ROS-mediated biphasic pulmonary inflammation is shown after inhalation exposure [130].
(d) Titanium dioxide nanoparticles (TiO_2_ NPs): Low acute toxicity but accumulate in the liver or lung with chronic exposure. Mechanisms:
  − MAPK pathways (p38, c-Jun N-terminal Kinase [JNK]) activation [110,111].
  − JAK2-signalling-mediated expression of interleukin-6 (IL-6) [131].
  − Caspase-3-dependent apoptosis [132,133,134,135].
(e) Silicon dioxide nanoparticles (SiO_2_ NPs): exhibit low acute toxicity; however, long-term pulmonary and hepatic retention results in an inflammatory cell influx [131,136].
(f) Nickel nanoparticles (Ni NPs) activate the JAK/STAT signalling axis, elevating IL-6, IL-8, interleukin-10 (IL-10), and tumour necrosis factor-alpha (TNF-α), thereby driving strong pro-inflammatory responses [112].

### 6.3. Carbon-Based Nanomaterials

Carbon nanotubes (CNTs): Toxicity is a matter of content and/or structure. Single-walled carbon nanotubes (SWCNTs) are found to be more cytotoxic than multi-walled carbon nanotubes (MWCNTs) due, among other factors, to their small diameter size and high reactivity [137,138]. Mechanisms:

− Involvement of the lungs, mainly via inhalation, including pulmonary inflammation.
− Production of ROS and disturbance in the function of mitochondria.
− PEGylation or functionalization partially mitigates toxicity.

### 6.4. Polymeric Nanoparticles

Polyethylene glycol (PEG) and poly(lactic-co-glycolic acid) (PLGA): Amphiphilic FDA-approved PEG or PLGA-based NPs are biodegradable and have low cytotoxicity with predictable degradation [16,17]. Safety profile:

− Controlled degradation hydrolytically to lactic acid and glycolic acid.
− Uncommon immune activation at large doses (activation of the complement pathway).
− High biocompatibility facilitates repeated and prolonged treatment.

### 6.5. Quantum Dots

Cadmium-based quantum dots (QDs): Some of the most toxic NPs due mainly to their leakage of Cd^2+^. Mechanisms:

− ROS accumulation.
− Apoptosis and mitochondrial dysfunction.
− Neuronal and epithelial cell DNA fragmentation [18,20,139,140].
− Surface passivation (e.g., polymer coatings) reduces the release of Cd^2+^, but not its toxicity.

### 6.6. Comparative Insights

The cytotoxicity of NPs should not be generalised and should be considered on a case-by-case basis, depending on their composition, size(s), surface characteristics, and exposure conditions.

Despite the biochemical variety, NP would appear to be toxic to shared molecular pathways:

(1) ROS-mediated oxidative stress (Ag, ZnO, TiO_2_, QDs), [102,110,111].
(2) Nrf2/HO-1 inhibition (ZnO, Ag, Au), [125,126,127,128,129].
(3) The induction of the MAPK pathway (ZnO, TiO_2_), [110,111].
(4) JAK/STAT pathway pro-inflammatory signalling (Ni, TiO_2_) [112,131,136].
(5) mTOR-regulated autophagy (Ag, ZnO, TiO_2_, Au) [121,122,123,139,140].
(6) Caspase-dependent apoptosis (Ag, ZnO, TiO_2_, QDs) [132,133,134,135,141,142].

Thus:

− Ag, ZnO, CuO, and MnO NPs → high acute toxicity.
− (TiO_2_, SiO_2_, Ni NPs) → Chronic inflammation effects.
− CNTs → inflammation, pulmonary fibrosis, granulomas.
− Polymeric NPs → the least toxic candidates for translation.
− QDs → do not because of heavy-metal toxicity.

As highlighted in Chapter 6, cytotoxicity is one of the most important translational hurdles in the clinical advancement of nanoparticle formulations. The biological impact of nanoparticles depends largely on particle type, size, shape, surface charge and chemistry. Many effects of metallic and oxide nanoparticles are related to the generation of reactive oxygen species (ROS), mitochondrial perturbation, apoptotic events, and stimulation of pro-inflammatory pathways. Polymeric nanoparticles, including PLGA, are generally biocompatible; however, based on the formulation, they can cause complement activation and immune elimination. Lipid nanocarriers, known to be used effectively for drug delivery, can induce inflammatory and cytotoxic effects in a dose-dependent manner. Additionally, carbon-based nanomaterials display these concerns, as well as genotoxicity.

To summarise this mechanistic understanding, Table 3 compiles a comparative review of the major cytotoxicity features among an array of nanoparticle classes and their association with the physical environment, biological outcomes, or translatable aspects. The organised summary emphasises the need for rational design and surface functionalisation approaches, combined with standardised safety testing, to prevent adverse effects and ensure reproducibility in pre-clinical and clinical applications.

Figure 2 also summarises hybrid nanomedicine approaches (native EVs, membrane-coated NPs, EV–NP hybrids, and genetic modification of EVs) to provide therapeutic solutions for the pivotal cytotoxicity and safety challenges revealed herein, with a single translational roadmap in alignment with comprehensive critical reflection.

### 6.7. Summary

The cytotoxic responses of NPs are greatly dependent on their composition and exposure conditions. The most significant acute toxicity, mediated through ROS and apoptosis, is observed in the cases of Ag, ZnO, CuO, and Cd-based QDs, while a chronic inflammation effect was found only for TiO_2_, SiO_2_, and Ni NPs. CNTs are a cause of pulmonary fibrosis, and polymeric NPs (PEG; PLGA) are the safest and most clinically approved.

Mechanistic alignment across ROS, MAPK, JAK/STAT, autophagy, and apoptosis suggests shared molecular nodal points with potential for targeted mitigation of toxicity.

Future progress requires:

(1) Standardised toxicological protocols across models.
(2) Incorporation of mechanistic biomarkers (ROS, cytokines, caspases) into regulatory matrices.
(3) AI-guided predictive toxicology to be added to experimental analysis and enhance reproducibility [23,24,92].

This integrated approach enables rational NP design, regulatory convergence, and safer translation into clinical practice.

## 7. Discussions

In this review, we put into perspective the dual promise and challenge of extracellular vesicles (EVs) or nanoparticles (NPs) in regenerative and personalised medicine. Although EVs offer a naturally existing platform with considerable promise in the safety realm, synthetic NPs provide flexibility in design and functionality; however, concerns will always linger regarding whether the foreign bodies are compatible with biology.

The trade-off between functionality and safety appears as a common thread throughout the literature. Metallic and metal oxide NPs, for example, show good antimicrobial and imaging results but at clinically applicable concentrations often cause oxidative stress and mitochondrial disruption. This is further supported by mechanistic studies demonstrating the overproduction of ROS, disruption of antioxidant systems (e.g., Nrf2/HO-1), and stimulation of apoptosis or autophagy. In contrast, polymer-based systems, such as PEG- and PLGA-nanocarriers, have low acute toxicity profiles, controlled degradation rates, and good biodistribution profiles, which is why these carriers are used in some FDA-approved products. However, even such “safer” platforms may induce immune activation at high doses or sequential administration.

Carbon-based nanomaterials and quantum dots comprise two other areas with significant translational potential, but they also pose significant risks. SWCNTs have been consistently observed to demonstrate pulmonary toxicity in inhalation studies, 22 whereas surface-functionalized MWCNTs are less reactive but still induce granulomatous responses. Cd-based QDs still have enormous application potential in imaging, but here they suffer from Cd-ion leakage and related ongoing concerns regarding DNA damage and neurotoxicity. These findings underscore the importance of incorporating mechanistic toxicological thinking into the early design of nanomaterials.

Notably, the comparative cytotoxicity analysis (Table 3) enables disparate observations to be integrated within a common framework, making risk portfolios across NP classes more visible. This incorporation ensures that data are often fragmented in the literature, and the scope is inconsistent. Through the unification of toxicity endpoints (ROS, apoptosis, autophagy, and inflammation) in different cell types and under varied exposure conditions, comparative integration suggests a more robust evidence base for regulatory deliberation and translational projects.

There are significant implications for regenerative and personalised medicine. In the context of therapeutic delivery, polymeric NPs offer biotherapeutics as a more clinically established/relevant option, with predictable pharmacokinetics and regeneratable production. On the other hand, metallic and oxide NPs may find applications in targeted imaging, local antimicrobial or combinational therapies, where their reactivity is beneficial but systemic exposure must be avoided. For instance, carbon-based nanomaterials and quantum dots are promising from a technological perspective, but will need significant refinement of surface functionalization or replacement with less toxic elements to circumvent existing safety thresholds.

The barriers to translation, as presented in Figure 2—namely, toxicity, immunogenicity, scalability, and regulatory challenges—continue to be the most significant bottlenecks limiting the broad clinical application of EV–NP hybrid technologies. Although creative engineering solutions have shown encouraging preclinical outcomes, addressing these obstacles will require technical improvements, as well as standardised manufacturing procedures, strict safety testing, and synchronised regulatory guidelines. Accordingly, the sketch in Figure 2 emphasises this challenge from bench to bedside.

Finally, standardisation is urgently needed. Differences in experimental models, exposure conditions and outcomes have led to a lack of comparability among studies. The addition of mechanistic biomarkers (ROS, caspases, Nrf2, MAPKs, and JAK/STAT signalling pathways) as well as the standardisation of protocols among different laboratories will further enhance reproducibility. New technologies, such as AI-based predictive toxicology and multi-omics integration, may complement classical assay methods, leading to earlier and more precise assessments of the risk potential of NMs.

In conclusion, the field has reached an inflexion point, and the future for both EVs (which exert innate safety) and NPs (that present unparalleled adaptability in chemical architecture and component makeup) critically depends on matching the science of function with safety via controlled toxicity assessment, mechanistic insight generation, and regulatory alignment.

## 8. Conclusions

Extracellular vesicles (EVs) and nanoparticles (NPs) have been pioneering tools in regenerative and personalised medicine, each offering unique advantages along with distinct challenges. EVs possess natural biocompatibility, intrinsic targeting, and intercellular communication, while NPs offer tunable design, reproducibility, and scalable fabrication processes. Their drawbacks—heterogeneity and poor yield (EVs) as well as cytotoxicity and immune recognition (NPs)—highlight the necessity of integrative approaches.

Hybrid and biomimetic strategies, such as membrane-coated carriers and EV–NP fusion platforms, are translationally appealing options that integrate biological specificity with engineering controllability. The concomitant emergence of AI reinforces this path, with potential predictive optimisation in design, toxicity assessment, and manufacturing standardisation. Such incorporation helps expedite the development of safe-by-design products and closes the gap between preclinical potential and clinical value.

The harmonisation of protocols and the use of mechanistic biomarkers for oxidation and inflammation, in line with international guidelines, is required to improve reproducibility between laboratories and facilitate regulatory acceptance. In so doing, innovation becomes compatible with safety, and personalisation can be reconciled with scalability, as EV-, NP- and hybrid-based therapies shift from experimental constructs to dependable clinical instruments.

If realised, these developments will revolutionise the treatment of degenerative, metabolic, and oncological diseases—rendering regenerative medicine a personalised, safe, and patient-focused field.

## Figures and Tables

**Table 1:** Comparative Analysis of EVs and NPs.

Feature	Extracellular Vesicles (EVs)	Nanoparticles (NPs)
**Immunogenicity**	Low immunogenicity due to natural composition; evade immune recognition [30,31]	Higher immunogenicity due to synthetic composition; often recognised as foreign particles [32,34]
**Biodistribution**	Natural tropism towards specific tissues; efficient crossing of biological barriers [35,36]	Biodistribution influenced by physical and chemical properties; rapid clearance by RES [32,37]
**Targeting Ability**	Inherent targeting ability based on cellular origin and surface proteins [36,38]	Targeting ability can be engineered through surface modifications [33,34]
**Therapeutic Efficacy**	High therapeutic efficacy due to efficient delivery and minimal immune interference [36,38]	Variable therapeutic efficacy due to challenges in targeting and immune clearance [32,39]

**Table 2:** AI in EV and NP studies.

AI Method	Application Area	Specific Contribution	Outcomes	References
Random Forest (RF), Support Vector Machine (SVM), Artificial Neural Network (ANN)	Nanoparticle toxicity assessment	Modelling cytotoxicity based on size, charge, and surface chemistry	Accurate prediction of toxicity; reduction in animal testing	[23,24,92]
Convolutional Neural Networks (CNNs)	EV-based diagnostics	Analysis of EV microstructural and morphological features	Accurate classification of breast cancer subtypes	[26]
Explainable AI (XAI)	EV biomarker interpretation	Multi-omics feature selection and interpretability	Improved prediction transparency; liver disease staging	[91]
Multi-omics integration with ML models	EV characterisation and functional profiling	Integration of proteomic and transcriptomic EV data	Improved reproducibility of EV-based therapy design	[25,28,95]
Deep Learning, Hybrid Models	Nanoparticle design and biodistribution	Prediction of NP–drug interactions, tumour targeting, and BBB penetration	Optimised formulations; accelerated oncology and neurology applications	[93]
AI-based formulation optimisation	Standardisation of NP production	Linking experimental conditions with NP quality parameters	Reduced variability; improved reproducibility across labs	[94]
Hybrid AI models	Protein corona and immune interactions	Predicts clearance and opsonisation	Improved stealth strategies	[95,96,97]

**Table 3:** Cytotoxicity of major nanoparticle classes.

NP Type	Size/Dose	Experimental Model	Observed Effects	References
Silver (AgNPs)	1.4–18 nm; 5–50 µg/mL	Hepatocytes, neuronal cells, rodents	ROS generation, mitochondrial damage, apoptosis (size-dependent)	[105,106,107,108,109,113,114]
Gold (AuNPs)	5–50 nm; chronic exposure	Rodent cortex, hippocampus	Bioaccumulation, altered signalling, neuroinflammation	[18,19,110,111,120,121,122,123]
Zinc oxide (ZnO NPs)	20–100 nm; >10 µg/mL	Hepatocytes, renal cells	ROS induction, mitochondrial dysfunction, apoptosis, and MAPK activation	[124,125,126,127,128,129]
Copper oxide (CuO NPs)	20–80 nm	Neuronal, epithelial cells	Strong ROS production, high cytotoxicity	[11]
Iron oxide nanoparticles Fe_3_O_4_/Fe_2_O_3_	10–100 nm	Pulmonary/liver models	Lysosomal accumulation, inflammation	[130]
Titanium dioxide (TiO_2_ NPs)	10–100 nm; high doses	Epithelial cell lines	Mild ROS production, low acute toxicity; risk of chronic accumulation	[110,111,112,131,132,133,134,135,136]
Silicon dioxide (SiO_2_ NPs)	20–200 nm	Lung, liver cells	Low acute toxicity; chronic accumulation	[131,136]
Single-walled carbon nanotubes (SWCNTs)	Diameter < 2 nm	Rodent lung models	Pulmonary inflammation, fibrosis, and ROS generation	[138]
Multi-walled carbon nanotubes (MWCNTs)	Diameter > 10 nm	Rodent lung models	Lower reactivity, fibrosis and granuloma formation	[138]
Polymeric NPs (PEG, PLGA)	50–200 nm	Multiple in vivo models	Minimal cytotoxicity, biocompatible degradation, and immune activation at high doses	[16,17]
Quantum dots (Cd-based QDs)	2–10 nm	Neuronal, epithelial cells	Cd^2+^ release, ROS, DNA damage	[18,20,139,140]

## Data Availability

No new data were created or analyzed in this study.
